# The Ecology and Human Biology of Pastoralists: Building on the Contributions of Michael A. Little

**DOI:** 10.1002/ajhb.70115

**Published:** 2025-08-04

**Authors:** William R. Leonard

**Affiliations:** ^1^ Department of Anthropology, Program in Global Health Studies Northwestern University Evanston Illinois USA; ^2^ Robert J. Havey, MD Institute for Global Health, Feinberg School of Medicine Northwestern University Chicago Illinois USA

**Keywords:** human biology of pastoralists, pastoralist, special issue

## Abstract

Human biologists have long studied the ecology, health, and adaptive patterns of pastoralist populations around the world. Over the last 20 years, research among pastoralists has increasingly focused on how ongoing climatic and socioeconomic changes are influencing these populations and threatening this lifeway. Additionally, with the development and broader use of “field friendly” methods for measuring energy expenditure, metabolism, and diverse biomarkers of physiological health, we are now able to gain a much more detailed and dynamic picture of the adaptive strategies of pastoralists. This Virtual Special Issue of the *American Journal of Human Biology*, “Human Biology of Pastoralists Populations” (Edited by Benjamin Campbell), showcases important advancements in this research domain and highlights the foundational contributions of Michael A. Little to our understanding of the biology and health of pastoralist societies.

## 1 Introduction

In this Virtual Special Issue of the *American Journal of Human Biology*, Guest Editor Ben Campbell and colleagues examine some of the major advancements in the study of the human biology of pastoralists over the last 20 years. This Special Issue is based on a Symposium from 2024 at the 49th Annual Meeting of the Human Biology Association, “Tribute to Michael Little: Last 20 Years of Human Biology Research among Pastoralists”. Each of the contributions to this special issue addresses and builds upon the foundational work that Dr. Little has carried out over the course of his career.

In this paper, I start by recognizing and discussing Michael Little's contributions to our understanding of the biology, ecology, and adaptability of pastoralist societies. I then discuss how each of the papers in this special issue advances our understanding of diverse aspects of the biology of pastoralists and how they are adapting to dramatic social and environmental changes.

## 2 Contributions of Michael A. Little to the Study of Pastoralists

The majority of Mike Little's career has been devoted to the study of pastoralists—first, with his early work with Quechua agropastoralists of the Peruvian Andes, and then beginning in the mid‐1970s, with the long‐term study of the Turkana of Northwest Kenya. Mike, more than anyone in our field, has played a dominant role in shaping research on the ecology and biology of pastoralist societies.

**FIGURE 1:**
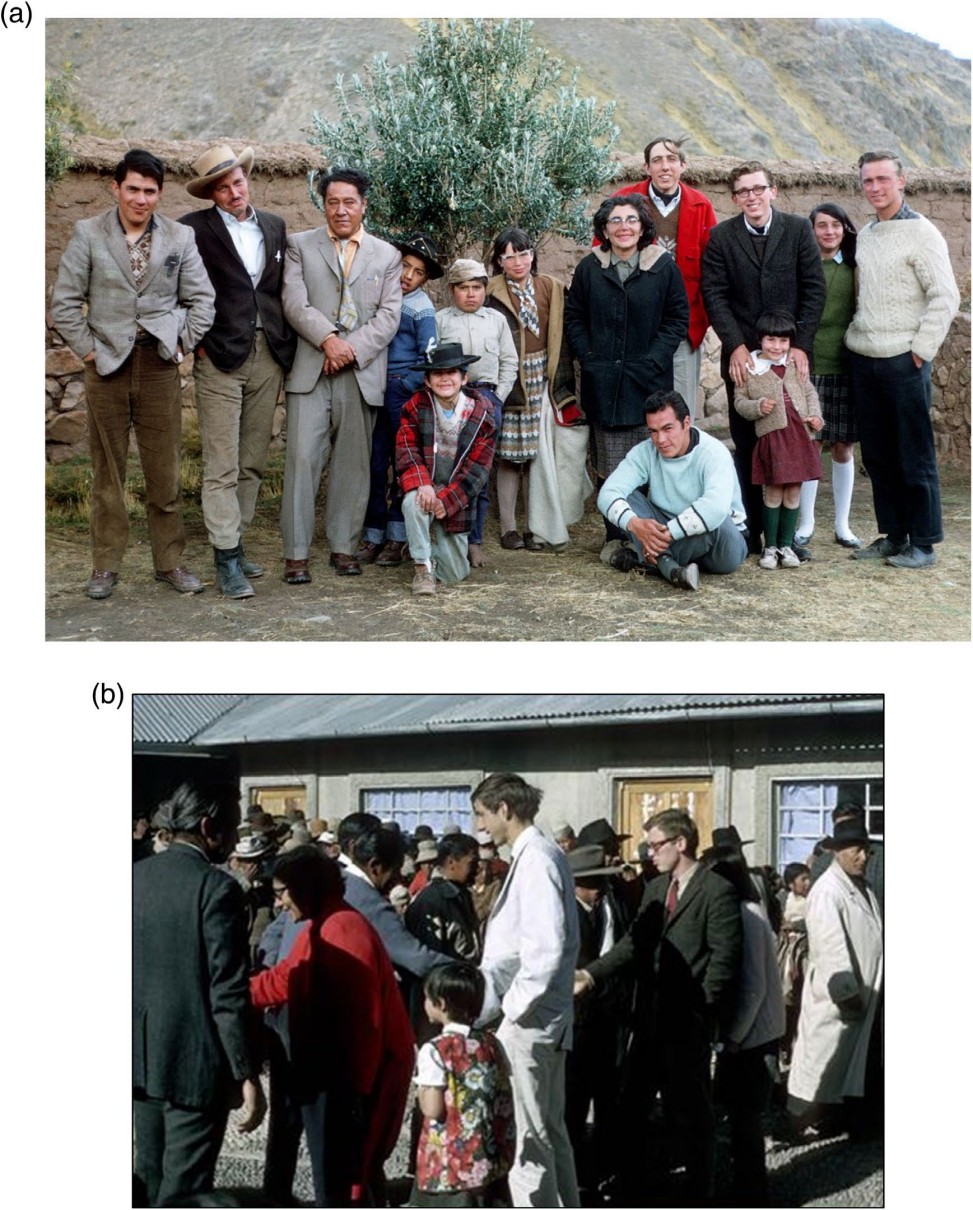
(a) The Penn State High Altitude Research Team, Nuñoa District, Peru, 1964. From left are: Roberto Frisancho, Paul Baker, Lucas Guerra, Jefferson Guerra, Joshua Baker, unidentified child, Amy Baker, Thelma Baker, Victor Barreda, Brooke Thomas, Michael Little, Felicia Baker, Deborah Baker, and Anthony Way. (b) Michael Little (glasses, dark jacket, and tie), along with Brooke Thomas (white jacket) at the dedication of the Penn State Research Lab in Nuñoa, Peru, 1965. Photos courtesy of Anthony Way.

Figure 1 shows photographs from Mike's early work as part of the Penn State High Altitude Research Group in the Peruvian *altiplano* in the 1960s and early 1970s. Figure 1a shows Mike, along with fellow graduate students Roberto Frisancho, Brooke Thomas, and Tony Way, and mentors Paul and Thelma Baker in Nuñoa, Peru in 1964. Figure 1b shows Mike, along with Brooke Thomas, at the dedication of the research lab in Nuñoa, where much of the early pioneering work on high altitude adaptation was carried out.

**FIGURE 2:**
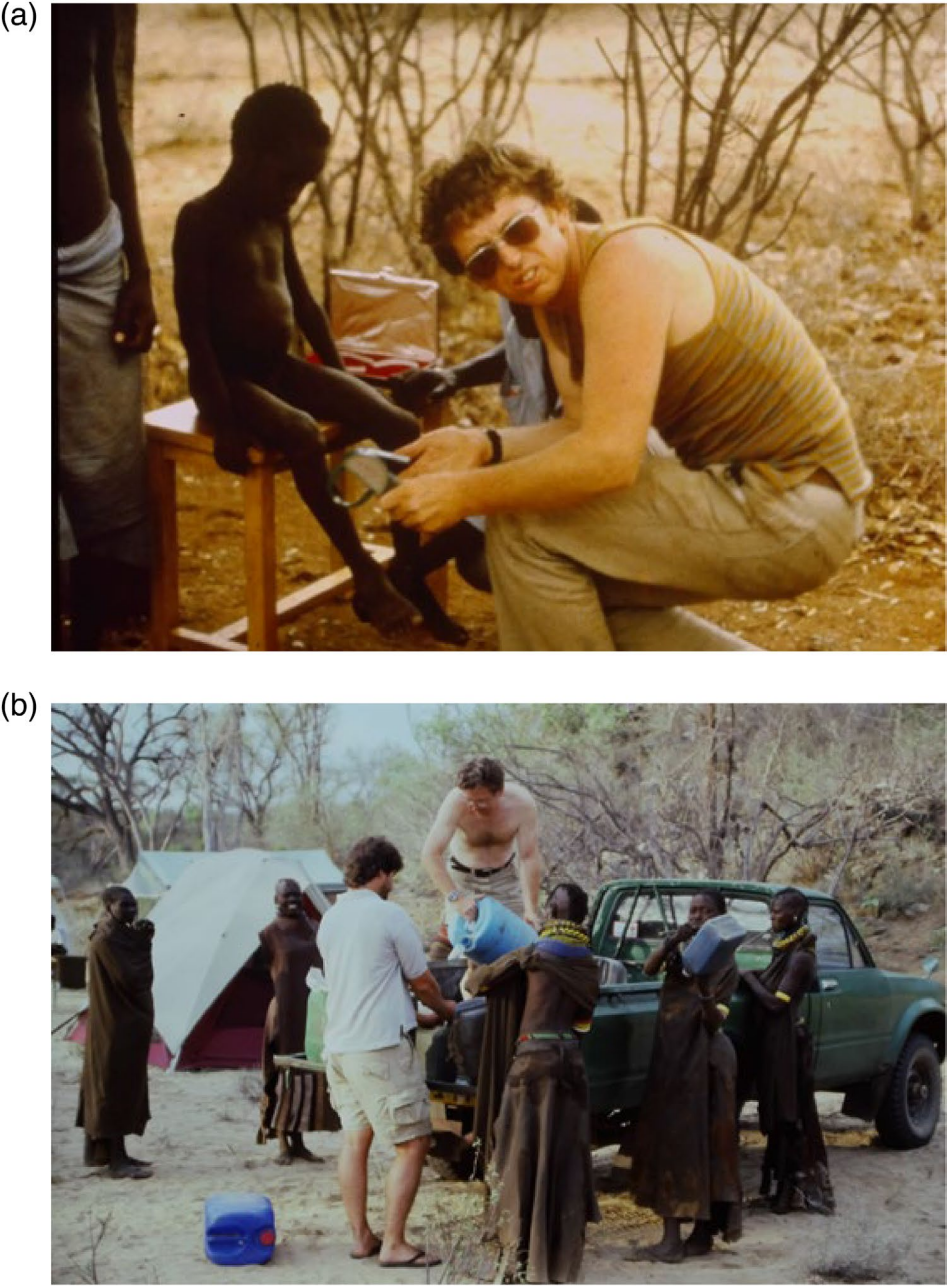
(a) Michael Little taking skinfold measurements on a Turkana child, 1986, and (b) providing water to Turkana women, 1990. Photos courtesy of Paul Leslie.

Figure 2 includes images from Mike's later work with the Turkana of Northwest Kenya. These show Mike collecting anthropometric data for the assessment of growth and nutritional status (Figure 2a) and distributing water to Turkana women (Figure 2b).

These research projects resulted in two of the landmark edited volumes in our field: (a) The *Man in the Andes* volume, co‐edited with Paul Baker, synthesizing the foundational high‐altitude work from the 1960s and early 1970s (Baker and Little 1976) and (b) *Turkana Herders of the Dry Savanna*, co‐edited with Paul Leslie, summarizing their long‐term research with the Turkana (Little and Leslie 1999).

Additionally, although less well‐known, Mike's 1976 book with George Morren, *Ecology, Energetics, and Human Variability*, is a gem (Little and Morren 1976). In this short book, Little and Morren lay out a framework for studying human biological diversity from an ecological and energetics perspective. This ecological‐energetics framework is the foundation of Mike's research on pastoralist populations, and it continues to shape our work today. This powerful perspective allows us to explore biological and behavioral adaptations/responses to subsistence in the marginal and low productivity ecosystems inhabited by pastoralist populations (see Thomas 1973, 1976; Coughenour et al. 1985; Little et al. 1990; Galvin 1992; Leonard 1991; Leonard et al. 1996, 2002).

The classic energy flow model developed from the high altitude research in Peru documented the “flows” (amounts) of dietary energy for Quechua households being derived from the herd animals (llama, alpaca, sheep), agriculture (tubers and cereal grains), and non‐local foods from lower elevations obtained through the market and exchange (see Little and Morren 1976; Little et al. 1990; Thomas 1973, 1976). This model provided important insights into the adaptive strategies of the highland Quechua. It documented that the Peruvian *altiplano* is a “low energy” ecosystem for humans that necessitated a number of distinctive bio‐behavioral responses. These responses included the vertical exchange of resources with populations from lower elevations; notably exchanging alpaca wool for calorically dense foods, allowing the population to meet its energy requirements (Thomas 1976; Little and Baker 1976). Additionally, the division of labor among the Quechua, allocating key subsistence tasks to children and adolescents rather than adults, allowed for critical subsistence work to be done with less energy (Thomas 1976; Little and Baker 1976; Leonard 1991).

**FIGURE 3:**
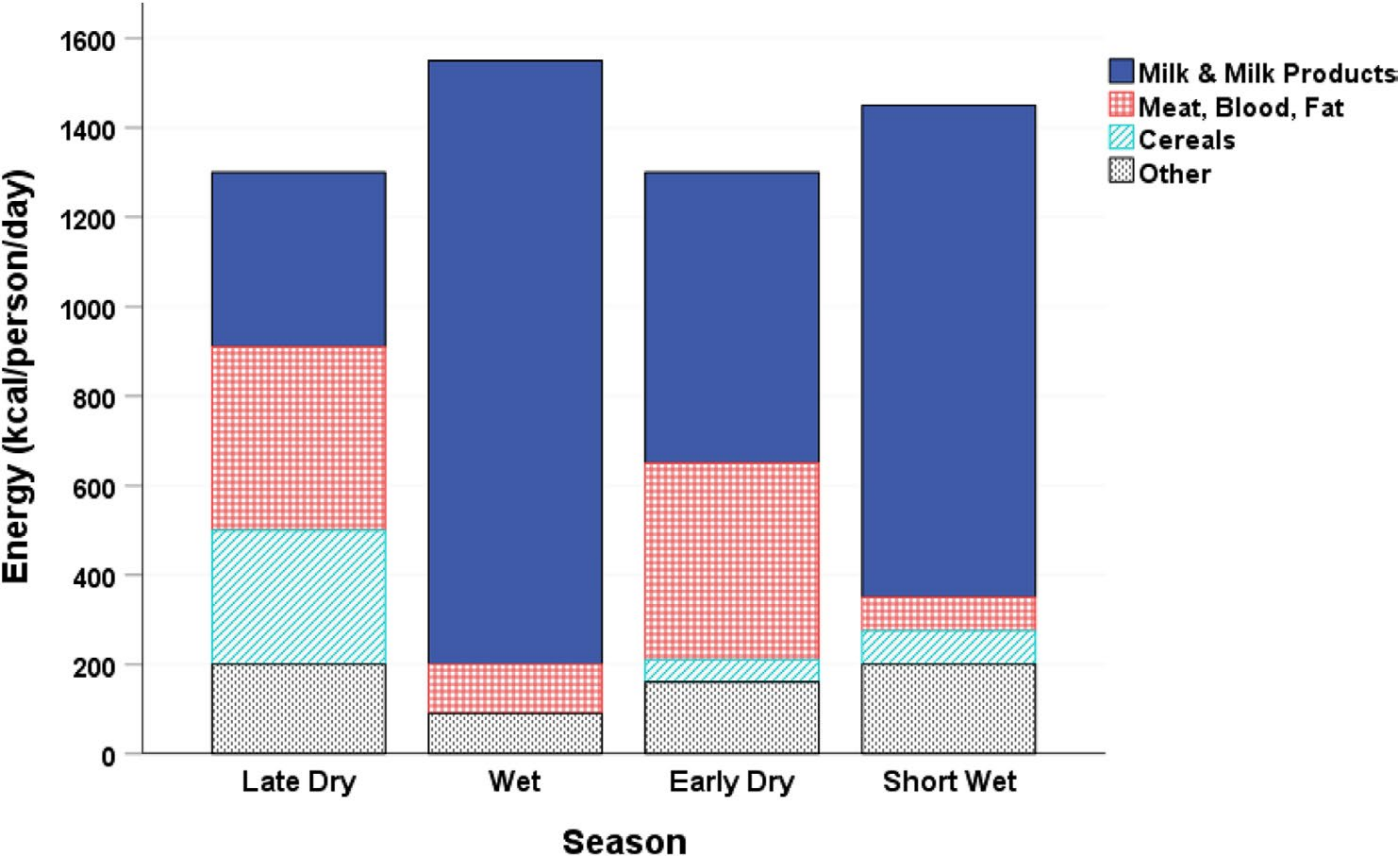
Seasonal variation in energy intake (kcal/person/day) and diet composition among Turkana households, 1981–1982. The composition of the diet varied markedly across the seasons. Across all seasons, milk contributed greater than 60% of dietary energy in the Turkana. *Source:* Data from Galvin (1985).

This same energetics approach was used by Little and colleagues to explore the subsistence ecology of the Turkana (see Coughenour et al. 1985; Galvin 1985, 1992; Gray 1994a; Galvin and Little 1999; Little 2002; Little et al. 1990). This work highlighted how the Turkana exploit multiple, diverse energy sources to sustain their population in a harsh and often unpredictable environment. Figure 3 shows the marked seasonal changes in daily energy intake (kcal/person/day) and the dietary energy sources (e.g., milk, meat and other animal products, cereal grains) that the Turkana exploit. The long‐term research program among the Turkana elegantly documented how seasonal and inter‐annual variation in energy availability and food resources shaped a tremendous range of human bio‐behavioral measures, including: (a) energy expenditure and activity patterns (Galvin 1985, 1992; Galvin and Little 1999), (b) physical work capacity (Curran‐Everett 1994; Curran and Galvin 1999), (c) patterns of physical growth and development (Little 1989; Little et al. 1983, 1999; Little and Johnson 1987; Little and Gray 1990), (d) variation in adult body size and nutritional status (Little and Johnson 1986; Galvin 1992; Pike 1999; Campbell et al. 2005), (e) immune status and infectious disease risks (Shell‐Duncan 1993, 1995; Shell‐Duncan et al. 1999), and (f) fertility and reproductive function (Leslie and Fry 1989; Gray 1994b; Pike 1999; Leslie, Campbell, et al. 1999; Leslie, Dyson‐Hudson, and Fry 1999; Campbell et al. 2006).

The ecological energetics framework pioneered by Mike Little has grown and developed greatly in human biology. With advancements in techniques for measuring energy expenditure and activity levels in field settings, we are now able to explore patterns of energy flow and their implications for human biology and health with much greater rigor and precision (Leonard 2012). Indeed, this powerful framework has direct implications for understanding the diverse aspects of the biology of pastoralist populations that are examined in each of the papers in this special issue.

## 3 Papers in This Special Issue

Ben Campbell's (2025) paper nicely highlights some of the major areas of expansion and change that we have seen with the study of pastoralists over the last 20 years. In studies of pastoralist populations around the world, there has been a re‐examination of adaptation to climatic stressors (i.e., heat, cold, high altitude) and their implications for energy metabolism, work capacity, and metabolic health. Additionally, the distinctive patterns of growth and development among pastoral groups are now being looked at in a broader comparative context, drawing on new analytic approaches. Human biologists are now exploring dimensions of the reproductive ecology of pastoralist societies with much more nuanced and sophisticated approaches. Additionally, the broader application of genetic and epigenetic techniques is transforming our understanding of the diverse adaptive strategies evident in pastoralist populations.

The paper by Cara Ocobock et al. (2025) examines the distinctive metabolic challenges faced by herding populations of the arctic. Circumpolar herding populations of Europe, Asia, and North America have elevated resting metabolic rates in response to their cold, marginal environments (Leonard et al. 2005, 2014; Ocobock et al. 2020; Snodgrass et al. 2005). These elevations in metabolic heat production are partly attributable to thyroid hormone metabolism (Leonard et al. 1999; Levy et al. 2013). Ocobock et al. (2025) document particularly high rates of energy expenditure and distinctive thyroid hormone profiles among female reindeer herders of Finland, suggesting important gender differences in physiological responses to arctic conditions.

This work also highlights how some of the ongoing changes in both lifeways and climate are impacting Indigenous populations of the north. With shifts in diet and activity, along with ongoing climate change, risks for chronic metabolic diseases are rising in many Indigenous northern groups.

Masako Fujita et al. (2025) provide a detailed examination of the multiple potential behavioral and physiological pathways used by Ariaal agropastoral women of Kenya to protect maternal and infant nutritional health during severe drought conditions. They specifically consider how variables such as land holdings, livestock sales, and child fosterage influence the mother's nutritional status and the nutritional content of her breastmilk.

The results are intriguing and indicate that land size was *negatively associated* with maternal nutritional status. It appears that greater agricultural work leaves breastfeeding women more vulnerable to drought. Women from households more heavily involved in farming were more likely to be underweight and deficient in vitamin A.

Selling cattle was found to be protective against maternal risks for underweight but was not associated with milk energy content. These findings suggest that maternal physiology is playing a critical role in protecting infants from conditions of nutritional stress. In this context, it would be interesting to also examine some of the other, non‐nutrient components of breast milk to see if there is evidence of maternal signaling of subsistence patterns and environmental conditions, as has been identified in some of the evolutionary medicine literature (e.g., Klein et al. 2018).

The papers by Kedir Teji Roba et al. (2025) and Bilinda Straight et al. (2025) highlight the limits of human adaptability to conditions of climate change and heat stress among pastoralist populations of northern Kenya. Drawing on research with the Daasanach, Roba et al. (2025) document the effects of the severe drought from 2020 to 2022 on water security, chronic stress, and inflammation among adult heads of households. They found that higher levels of stress and greater water insecurity were both significantly associated with higher levels of inflammation, as measured by C‐reactive Protein (CRP) levels. This work demonstrates the multiple pathways through which extreme climatic events impact human health and well‐being among herding populations. Further, the findings underscore the critical need for investments in safe and reliable water sources to promote resilience in these communities.

Straight et al. (2025) examine epigenetic changes linked to in utero exposure to heat stress among the Samburu. They have found differential methylation in genes associated with stature, tibial growth, and immune function based on heat exposure in the first trimester in utero. These findings shed new light on adaptive pathways for responding to climatic stressors, as this is the first study to identify biological mediators linking environmental heat to lower limb growth in children. Moreover, this study offers a potential epigenetic mechanism for explaining long‐documented ecogeographic variation in human body proportions. Indeed, this work provides an intriguing explanation for Allen's Rule, as it applies to human populations (see Roberts 1978; Katzmarzyk and Leonard 1998).

## 4 Conclusions

Overall, a number of important themes emerge from this impressive set of papers. First, is the demonstration of the remarkable range of biological, behavioral, and social responses that pastoralist populations use to adapt to some of the most challenging and unpredictable ecosystems on the planet. This research offers a dynamic picture of how pastoralist societies are adjusting to ever‐changing landscapes.

Additionally, I am struck by how the application of novel methods of both data collection and analysis are enriching and transforming our understanding of the human biology of pastoral groups. These include the development of “field friendly” methods for measuring energy expenditure, metabolism, and physiological health; the tremendous expansion of genetic and epigenetic analyses; and the application of new quantitative and statistical approaches to better explore patterns of variation within and across populations.

Finally, all of us who work with pastoralist populations are recognizing the dramatic impact that climate change is having on these groups—making environments more unpredictable and stretching the limits of human resiliency and adaptive potential.

Yet, while there are clearly many ecological, social, and economic forces that threaten pastoralist populations around the world, I am encouraged by the thoughtful and rigorous work being done by human biologists to better understand these problems and inform the development of productive interventions and solutions. I see this as a fitting tribute to the foundational research done by Michael A. Little!

## Data Availability

Data sharing is not applicable to this article as no new data were created or analyzed in this study.
